# Supplementary material for: Sir2 is required for Clr4 to initiate centromeric heterochromatin assembly in fission yeast
Source: EMBO J. 2013 Jun 14;32(17):2321–35. doi: 10.1038/emboj.2013.143 (PMC3770337; doi:10.1038/emboj.2013.143)
Supplement: Supplementary Data [file emboj2013143s1.pdf]

Supplementary Information.

Supplementary Methods:

Strain construction

Integration of *sir2*$^+$-*his3*$^+$ and *sir2N247A-his3*$^+$ was performed following digestion of JP1267 and JP1312 with NruI and transformation of PY4765. Integrants were selected by growth on media lacking histidine, and southern analysis to ensure single copy integration.

TAP tagging of Sir2 and sir2N247A was performed by homologous recombination across C terminal sequences of *sir2*$^+$ with PCR product generated from a pFA6aTAP-NatR vector (JP1290) with homology to the *sir2* ORF and 3' UTR sequences. The tagged *sir2* locus was checked by sequencing, and strains were outcrossed twice prior to analysis.

clr4$^+$ reintroduction into the genome was performed using JP1326 which was digested with HpaI. Integrants were selected for growth on media lacking adenine, and single copy integration was confirmed by southern analysis.

The 3xFlag epitope was introduced between the ATG of *clr4*$^+$ and the 2nd codon by homologous recombination, in a strain where *ura4*$^+$ was inserted at that site (Py1249) to generate Py1664 following selection on FOA, sequencing of the locus, and outcrossing.

Plasmid construction

All PCR-generated cloned fragments were confirmed by sequence analysis. Constructs for expression of GST fusion proteins were generated by amplification of *sir2* cDNA sequences with primers bearing EcoR1 restriction sites, and cloned into EcoR1 digested pGEX-GK (Guan and Dixon, 1991).

Plasmids for integration of wild type or N247A mutant Sir2 linked to *his3*$^+$ (JP1267 and 1312) were generated by PCR amplification of genomic *sir2*$^+$ with primers containing Not1 and Pst1 restriction sites, and cloned into Not1/ Pst1 digested JP1142, which is pRO319 (Adams *et al.*, 2005) that has been converted to an integration vector by AatII digestion to remove ARS sequences. Mutagenesis of *sir2N247A* was performed by PCR with a mutagenic primer incorporating a 5' Sph1 site, and with the 3' Pst1 primer. The N247A mutation was incorporated into JP1312 by digestion of JP1267 with Pst1 and Sph1 and replacement with the mutant PCR product.

A TAP tagging cassette linked to NatR was generated by subcloning NatR from pFA6a NatMX6 (Bahler *et al.*, 1998;Van *et al.*, 2005) by NcoI/ SacI digestion to replace KanR in pFA6a TAP-KanR (Tasto *et al.*, 2001) (plasmid 2021 from K. Gould).

The *sir2* expression construct JP1613 was generated by PCR of Sir2 cDNA with primers bearing SalI and BamHI sites, which did not include the stop codon. The PCR fragment was cloned into XhoI and BglII sites of JP1611, which is a C terminal 3xV5 tagging vector in a pREP81 backbone. JP1611 was generated by release of the 3xV5 tag from pSLF972 (kind gift

from Susan Forsburg) through XhoI, SacI digestion and recloning of the 3xV5 tag into XhoI, SacI digested JP802 which is a derivative of pREP81.

The plasmid used for reintegration of *clr4*$^{+}$ linked to *ade6*$^{+}$ at single copy into the genomic locus following linearization with HpaI (JP1326) was derived from JP1084 (Partridge *et al.*, 2007). The *his3*$^{+}$ marker was released by FseI digestion and replaced with *ade6*$^{+}$ from pRO317 (Adams *et al.*, 2005).

Plasmids for episomal expression of genomic *3xFlag-clr4*$^{+}$ linked to *his3*$^{+}$ (JP1636) and *leu1*$^{+}$ (JP2100) were generated by PCR amplification of the *3xFlag-clr4*$^{+}$ locus from Py1664 with primers incorporating SacII and SalI restriction sites (used previously to amplify *clr4*$^{+}$, Partridge et al 2007), and were cloned into SacII/ SalI sites of JP1049 (pR0319-*his3*$^{+}$ (Adams *et al.*, 2005) or JP1050 (pRO320-*leu1*$^{+}$ (Adams *et al.*, 2005). Mutagenesis of JP1636 was performed by in phusion PCR to generate H410K mutant of genomic *3xFlag-clr4*$^{+}$ (JP2111).Plasmids for episomal expression of genomic *clr4*$^{+}$ (JP1078 and empty vector JP1045) have been described previously (Partridge *et al*., 2007)

Fluorogenic peptide deacetylation assay

A 25 μL deacetylase reaction was assembled comprising 0.8 μM GST, GST-Sir2, or GST-Sir2N247A, 5mM Fluor-de-lys green substrate, 8mM NAD^{+} and 1mM histone peptide where applicable in a reaction buffer of 50 mM Tris pH 8.0, 100 mM NaCl, and 1 mM DTT. Enzymatic reactions were performed for 3 h while dark at ambient temperature (22º C). Termination of the enzymatic reaction was achieved by addition of 25 μl quenching and developing mixture comprising 0.25 μL 2 mM trichostatin A (10 μM final in quenched reaction mixture), 0.5 μL 50 mM Nicotinamide (0.5 mM), and 1.25 μL trypsin-based developer concentrate to the 25 μL enzymatic reaction mixture. Endpoint fluorescence was measured 15 min after addition of developer using a Synergy HT BioTek Scanner (485 nm excitation/520 nm emission). A standard curve was subsequently constructed by serial dilution of the deacetylated fluorogenic substrate, and was used to equate experimentally observed fluorescence units with molar quantities of substrate deacetylated.

^{32}P NAD^{+} hydrolysis assay for Sir2 deacetylase activity

0.2 µM affinity-purified GST, GST-Sir2, and GST-Sir2N247A were coincubated overnight at ambient temperature (22ºC) with 2.5 µCi ^{32}P NAD^{+} (ARC 0141, American Radiolabeled Chemicals Inc.; specific activity 800 Ci/mmol) in the presence of 0.1 µg/uL calf thymus histones (Sigma Life Sciences) or 0.5 mM acetylated or unacetylated histone $H3_{1\text{-}19}$ or $H4_{1\text{-}19}$ N-terminal peptides (Hartwell Center, St Jude Children's Research Hospital), in a 10 µL reaction mixture buffered with 50 mM Tris pH 8.0, 100 mM NaCl, 1 mM DTT. Following the coupled ^{32}P NAD^{+} hydrolysis/substrate deacetylation reaction, products were diluted 50-fold, and 3 µL of this mixture was resolved by reverse-phase TLC (LKSD silica gel 60 A, Whatman Inc.; mobile phase of 80% EtOH, 20% 2.5 M ammonium acetate). The relative mobility of hydrolyzed radioligands was assessed by autoradiography, and the extent of ^{32}P NAD^{+} hydrolysis product evolution was determined by quantitative densitometry (UN-SCAN-IT gel™ Version 6.1 gel analysis and graph digitizing software, Silk Scientific Inc.).

Supplementary References

1. Adams C, Haldar D, and Kamakaka RT (2005) Construction and characterization of a series of vectors for Schizosaccharomyces pombe. *Yeast* **22:** 1307-1314.

2. Bahler J, Wu JQ, Longtine MS, Shah NG, McKenzie A, III, Steever AB, Wach A, Philippsen P, and Pringle JR (1998) Heterologous modules for efficient and versatile PCR-based gene targeting in Schizosaccharomyces pombe. *Yeast* **14:** 943-951.

3. Guan KL and Dixon JE (1991) Eukaryotic proteins expressed in Escherichia coli: an improved thrombin cleavage and purification procedure of fusion proteins with glutathione S-transferase. *Anal Biochem* **192:** 262-267.

4. Partridge JF, Debeauchamp JL, Kosinski AM, Ulrich DL, Hadler MJ, and Noffsinger VJ (2007) Functional separation of the requirements for establishment and maintenance of centromeric heterochromatin. *Mol Cell* **26:** 593-602.

5. Tasto JJ, Carnahan RH, McDonald WH, and Gould KL (2001) Vectors and gene targeting modules for tandem affinity purification in Schizosaccharomyces pombe. *Yeast* **18:** 657-662.

6. Van DB, Tafforeau L, Hentges P, Carr AM, and Vandenhaute J (2005) Additional vectors for PCR-based gene tagging in Saccharomyces cerevisiae and Schizosaccharomyces pombe using nourseothricin resistance. *Yeast* **22:** 1061-1068.

Supplementary Figures

Figure S1. De-novo silencing defects in *sir2* deficient cells correspond with loss of centromere function. A. *Increased incidence of lagging chromosomes in late anaphase upon clr4$^+$ reintroduction in sir2Δ clr4Δ cells.* Yeast strains were fixed, stained with anti-tubulin antibodies, and DAPI. The percentage of cells with visibly lagging chromosomes was determined among late anaphase cells. Double-blind experiments were conducted in duplicate, with N≥200 for each strain background indicated**.**

Figure S2. De-novo silencing defects in *sir2Δ clr4Δ* to *clr4$^+$* cells are not due to defective clr4 transcription, and can be complemented by re-expression of *sir2$^+$* in *clr4$^+$* reintegrant backgrounds. A. *clr4$^+$ transcript levels are not decreased following reintegration of clr4$^+$ into sir2Δclr4Δ backgrounds*. Quantitative real time PCR of *clr4$^+$* transcripts relative to the euchromatic control, *adh1$^+$*, to monitor effects of reintegration of *clr4$^+$* into various mutant backgrounds. Data represent SEM of 2 biological replicate experiments.**B.** *Re-expression of sir2$^+$ in clr4$^+$ reintegrant strains complements the heterochromatic silencing defect*. Yeast strains bearing the centromeric *ura4$^+$* reporter were transformed with vectors expressing the V5 tag alone, or Sir2-V5. Serial dilution spotting assays were performed on media lacking leucine (to maintain selection for plasmids), and selective for growth of *ura4* expressing (-ura) or *ura4* silenced cells (+FOA). **C.** *Expression of Sir2-V5 was confirmed by western analysis.* Western analysis of extracts prepared from cells described in (A), using antibodies that recognize the V5 tag, or tubulin as a loading control.

Figure S3. De-novo silencing defects in *sir2Δ clr4Δ* to *clr4$^+$* cells are epigenetically heritable. Where indicated, yeast strains bearing the *ura4$^+$* centromeric transgene were restruck on nonselective medium, cultured for a time period corresponding to approximately 100 cell doublings, and assayed for growth by serial dilution spotting assays on nonselective (complete) medium and medium selective against uracil auxotrophy (+FOA).

Figure S4. GST-Sir2, but not GST-Sir2N247A or GST alone, promotes deacetylation of acetylated peptide substrates in vitro, and H4K16Ac is slightly increased in vivo in sir2D cells. *GST-Sir2 promotes the evolution of higher mobility ^{32}P NAD$^+$ hydrolysis products upon coincubation with radiolabeled cofactor, exclusively in the presence of acetylated peptide substrates***.** Representative autoradiographs following TLC separation of reaction products after coincubating **A.** GST alone, **B.** GST-Sir2, or **C.** GST-Sir2N247A with the substrates indicated in the presence of ^{32}P NAD$^+$, hydrolysis of which is required for coupled substrate deacetylation by Sir2 and its homologs. Relative evolution of higher mobility ^{32}P NAD$^+$ hydrolysis products was determined by quantitative densitometry, as presented in Figure 5E**. D.** *Western analysis of H4K16Ac in WT and sir2Δ cells, using a H4K16G mutant strain as control for antibody specificity.* Relative levels of the immunoreactive species indicated were quantified by densitometry. Experimental data presents the average of 2 experimental replicates from each of 2 distinct biological samples.

Figure S5. Centromeric transcript accumulation in H3K4A and H3K4Q mutants; H3K4Q mutation does not abolish de-novo centromeric silencing. *H3K4Q mutation produces significantly elevated centromeric dh transcript accumulation* **(A.)***, while accumulation of centromeric dg transcripts is comparatively attenuated* **(B.)***.* mRNA transcripts were evaluated by qRT-PCR amplification of cDNA and normalized to transcript levels of the *act1*$^{+}$ euchromatic control. Data presents the average of 2 experimental replicates. **C.** *H3K4Q mutation does not abolish de-novo centromeric silencing.* mRNA transcripts were evaluated by qRT-PCR as in panels A and B, but with 3 distinct biological replicates for the *K4Q clr4Δ to clr4*$^{+}$ reintroduction strain only.

Figure S6. Heterochromatin establishment at centromeres appears defective in H3K14A mutant cells. Quantitative PCR assessment of *dh* transcripts relative to *adh1*$^{+}$ following reintegration of *clr4*$^{+}$ into the *clr4Δ* genomic locus of H3K14A mutant cells. *clr4*$^{+}$ reintegration into single copy H3/H4 *clr4Δ* cells resulted in suppression of centromeric transcription, whereas transcripts remained high following reintegration of *clr4*$^{+}$ into *clr4Δ*H3K14A mutant cells. Note that transcript levels are also strongly elevated in H3K14A mutant cells under maintenance conditions.

Figure S7. Episomal genomic *Flag-clr4*$^{+}$ expression compensates for *clr4* function at centromeres and centromeric heterochromatin assembly is defective in histone H3 mutant backgrounds. A. *Plasmid based Flag-clr4*$^{+}$ *overexpression overcomes centromeric silencing defects in clr4Δ mutants.* Serial dilutions of the indicated strains each bearing the centromeric *ura4*$^{+}$ transgene were assayed for growth on medium lacking histidine (-His), as well as medium lacking histidine and uracil (-His -Ura), and medium lacking histidine and containing 5-fluoro-orotic acid (-His +FOA). **B.** *Swi6 does not associate with centromeres in H3K14A mutant background.* ChIP for Swi6 reveals a similar loss of recruitment of Swi6 to centromeric repeats (*dh*) in H3K14A and *clr4Δ* backgrounds. **C.** *Expression of Flag-Clr4 is not reduced by H3K14R mutation.* Protein extracts were made from single copy H3/H4 strains expressing WT or H3K14R mutant that were transformed with episomal Flag-Clr4. Western analysis revealed that levels of Flag-Clr4 were slightly upregulated in H3K14R cells compared with wild type, when normalized to the tubulin loading control. **D.** *Anti-Flag ChIP reveals a defect in Flag-Clr4 recruitment to centromeres in H3K14R mutant strains*. Anti-Flag ChIP was performed on WT or H3K14R histone mutant strains transformed with Flag or Flag-Clr4 expression vectors, and analyzed by quantitative real time for enrichment of centromeric *dh* and euchromatic *adh1*$^{+}$ sequences.

Figure S8. Catalytic mutant clr4H410K is stably expressed but is not enriched at centromeres. A. *Flag-clr4H410K is expressed as a stable protein.* Western blot against-epitope tagged Flag-Clr4 and Flag-clr4H410K provides evidence for specific detection of an immunoreactive species of the expected molecular mass. Denaturing protein extracts were prepared from equivalent cell inputs, resolved by SDS-PAGE and probed using antibodies against the Flag epitope. This blot was stripped and reprobed with a tubulin specific antibody, providing a loading control. Plasmid based overexpression of Flag-Clr4 and Flag-clr4H410K were driven by the genomic *clr4* promoter. **B.** *Flag-clr4H410K is not enriched at centromeres.* Q-PCR analysis of ChIP for Flag-Clr4 or Flag-clr4H410 enrichment (in otherwise *clr4Δ* cells) at

centromeric *dh* sequences compared with *adh1*$^+$ euchromatic control. Data averaged from 3 experimental replicates, with SEM shown.

Figure S9. Sir2 deletion does not reduce Clr4 levels or expression from the endogenous *clr4*$^+$ locus. A. *Sir2 deletion does not reduce detectable levels of Flag-Clr4 when expression is driven from the endogenous clr4*$^+$ *locus.* Anti-Flag immunoprecipitated samples were prepared from cell lysates normalized to contain equal amounts of total protein, resolved by SDS-PAGE, and probed by western blot against the Flag epitope. Crude lysates were also resolved by SDS-PAGE and probed by western blot against tubulin, to provide a loading control for sample input to the immunoprecipitation experiment. Relative levels of the immunoreactive species indicated were subsequently quantified by densitometry. Experimental data presents the average of 3 experimental replicates from each of 2 distinct biological samples. **B.** *Sir2 deletion does not reduce steady state levels of Flag-clr4*$^+$ *mRNA transcripts when expression is driven from the endogenous clr4*$^+$ *locus.* qRT-PCR amplification of random primed cDNA from yeast strains of the genotype indicated was performed, and *clr4*$^+$ transcript levels were normalized to transcript levels of the *adh1*$^+$ control. Experimental data presents the average of 2 experimental replicates**. C.** *Sir2 deletion does not impact clr4*$^+$ *transcript levels when it is expressed from genomic plasmid*. qRT-PCR analysis of random primed cDNA from indicated yeast strains. *clr4*$^+$ transcript levels were normalized to *adh1*$^+$, and expression of *clr4*$^+$ from genomic *clr4*$^+$ vector in *clr4Δ* cells was set to 100. Data represents average and SEM for 2 experimental replicates, with replicate experiments performed on 2 independent transformants for the triple deletion strain (n=4).

Table S1

Strain	Genotype	Figure
Py 2036	h- otr1R (Sph1)::ura4 ade6-210 leu1-32 ura4-DS/E	1,2,3,4,S1-S3,S9
Py 1838	h- clr4D::KanR otr1R (Sph1)::ura4 ade6-210 leu1-32 ura4-DS/E his3D	1,2,3,4,S1-S3,S9
Py 4765	h- sir2D::kanMX6 otr1R(SphI)::ura4 ura4-DS/E ade6-210 leu1-32 his3D	1,2,3,4,S1,S4
Py 5001	h- sir2-TAP-NAT otr1R Sph1::ura4 ura4-DS/E ade6-210 his3D leu1-32	1
Py 5008	h- sir2N247A-TAP-NAT otr1R Sph1::ura4 ura4-DS/E ade6-210 arg3D his3D leu1-32	1
Py 2971	h- clr4D::KanR otr1R Sph1::ura4 ade6-210 leu1-32 ura4-D18 his3D	2,3
Py 4795	h- sir2-his3+ otr1R(SphI)::ura4 ura4-DS/E ade6-210 leu1-32 his3D	1,2,4
Py 4820	h- sir2N247A-his3+ otr1R(SphI)::ura4 ura4-DS/E ade6-210 leu1-32 his3D	1,2,4
PY 4974	h- JP1326 ade6+ genomic Clr4 (HpaI)::clr4D::KanR otr1R Sph1::ura4 ade6-210 leu1-32 ura4-DS/E his3D	3,4,S1,S2
Py 4769	h- sir2D::kanMX6 clr4D::KanR otr1R(SphI)::ura4 ura4-DS/E ade6-210 leu1-32 his3D	3,4,S1,S2
Py 5090-5091	h- sir2D::kanMX6 JP1326 ade6+ genomic Clr4 (HpaI)::clr4D::KanR otr1R(SphI)::ura4+ ura4-DS/E ade6-210 leu1-32 his3D	3,S1,S2,S3
Py 2941	h- clr3Δ::kanMX6 his3+ leu1-32 ade6-210 otr1R(Sph1)::ura4 ura4-DS/E	2
Py 5088-5089	h- sir2D::kanMX6 clr3D::kanMX6 his3D leu1-32 ade6-210 otr1R(Sph1)::ura4+ ura4-DS/E	2
Py 8219-8220	h- sir2N247A-his3+ clr3Δ::kanMX6 his3D leu1-32 ade6-210 otr1R(Sph1)::ura4+ ura4-D18 or DS/E	2
Py 8221-8222	h- sir2+-his3+ clr3Δ::kanMX6 his3D leu1-32 ade6-210 otr1R(Sph1)::ura4+ ura4-D18 or DS/E	2
Py 4934	h- sir2+-his3+ clr4Δ::kanR otr1R(SphI)::ura4+ ura4-DS/E ade6-210 leu1-32 his3D	4
Py 4978-4979	h- sir2-his3+ JP1326 ade6+ genomic Clr4 (HpaI)::clr4D::KanR otr1R Sph1::ura4 ade6-210 leu1-32 ura4-DS/E his3D	4
Py 4929	h- sir2N247A-his3+ clr4D::kanR otr1R(SphI)::ura4 ura4-DS/E ade6-210 leu1-32 his3D	4
Py 4976-4977	h- sir2N247A-his3+ JP1326 ade6+ genomic Clr4 (HpaI)::clr4D::KanR otr1R Sph1::ura4 ade6-210 leu1-32 ura4-DS/E his3D	4
Py 7062-7063	h- H3.1/H4.1::his3+ H3.3/H4.3::arg3+ ade6-210 ura4-D18? his3D1 arg3D1	6,S5,S7
Py 6344-6345	h- H3.1/H4.1::his3+ H3.3/H4.3::arg3+ clr4D::KanR ade6-210 arg3D his3D leu1-32? ura4-DS/E?	6,S5,S6,S7
Py 1743	h- H3.2K9A H3.1/H4.1::his3+ H3.3/H4.3::arg+ leu1-32 Ura-D18 his3D arg3D	6

Py 6371-6372	h- H3.2K14A H3.1/H4.1::his3+ H3.3/H4.3::arg3+ ade6-210 leu1-32 his3D arg3D ura4D-18 OR ura4-DS/E	6,S6,S7
Py 7185	h- H3.2K4A H3.1/H4.1::his3+ H3.3/H4.3::arg3+ leu1-32 his3D arg3D ade6-210 (ura4-D18?)	6,S5
Py 6529	h- H4K16G H3.1/H4.1::his3+ H3.3/H4.3::arg3+ leu1-32 ura4D18 his3D arg3D ade6-210	6,S4
Py 6484	h- H3.1/H4.1::his3+ H3.3/H4.3::arg3+ JP1326 ade6+ genomic Clr4 (HpaI)::clr4D::KanR ade6-210 leu1-32? arg3D his3D	6,S5,S6
Py 7227	h- H3.2K4A clr4D::kanR H3.1/H4.1::his3+ H3.3/H4.3::arg3+ his3D arg3D ade6-210 leu1-32? ura4-D18 or DS/E	6
Py 7273-7276	h- H3.2K4A H3.1/H4.1::his3+ H3.3/H4.3::arg3+ JP1326 ade6+ genomic Clr4 (HpaI)::clr4D::KanR his3D arg3D ade6-210 leu1-32? ura4-D18/ DS/E?	6
Py 6514	h- H4.2K16G H3.1/H4.1::his3+ H3.3/H4.3::arg3+ clr4D::kanR leu1-32 ura4D18 his3D arg3D ade6-210	6
Py 6609-6610	h- H4.2K16G H3.1/H4.1::his3+ H3.3/H4.3::arg3+ JP1326 ade6+ genomic Clr4 (HpaI)::clr4D::KanR leu 1-32 ura4D18 his3D arg3D ade6-210	6
Py 8027-8028	h- H3.1/H4.1::his3 H3.3/H4.3::arg3 clr4D::KanR ade6-210 arg3D his3D leu1-32 ura4-DS/E? + episomal JP1050 [leu1+]	6,S7
Py 8030-8031	h- H3.1/H4.1::his3 H3.3/H4.3::arg3 clr4D::KanR ade6-210 arg3D his3D leu1-32 ura4-DS/E? + episomal JP2100 [genomic 3xFlag-Clr4 leu1+]	6,S7
Py 8036-8038	h- H3.2K14A H3.1/H4.1::his3+ H3.3/H4.3::arg+ clr4D::KanR ade6-210 leu1-32 his3D arg3D ura4D-18 OR ura4-DS/E + episomal JP2100 [genomic 3xFlag-Clr4 leu1+]	6
Py 8045-8047	h- H3.2K9A H3.1/H4.1::his3+ H3.3/H4.3::arg+ clr4D::KanR leu1-32 Ura-D18or DS/E his3D1 arg3D + episomal JP2100 [genomic 3xFlag-Clr4 leu1+]	6
Py 5557	h- JP1045 (empty vector, his3+) in clr4Δ::KanR ade6-210 leu1-32 ura4DS/E arg3D his3D	7,S9
Py 5517	h- JP1078 (genomic clr4+, his3+) in clr4Δ::KanR ade6-210 leu1-32 ura4DS/E arg3D his3D	7,S9
Py 8273	h- JP1045 (empty vector, his3+) in dcr1Δ::KanR clr4Δ::KanR ura4DS/E leu1-32 ade6-210 arg3? his3D	7,S9
Py 5522	h- JP1078 (genomic Clr4, his3+) in dcr1Δ::KanR clr4Δ::KanR ura4DS/E leu1-32 ade6-210 arg3? his3D	7,S9
Py 8266	h- JP1045(empty vector, his3+) in clr4Δ::KanR sir2Δ::KanR dcr1Δ::KanR his3D leu1-32	7,S9
Py 8263-8264	h- JP1078 (genomic clr4+, his3+) in clr4Δ::KanR sir2Δ::KanR dcr1Δ::KanR his3D leu1-32	7,S9
Py 6120	h- JP1611 [pREP81 Leu2+ C-term V5] in otr1R Sph1::ura4+ ade6-210 leu1-32 ura4-DS/E his3+ arg3+	S2
Py 6158	h- JP1611 [pREP81 Leu2+ C-term V5] in clr4Δ::KanR otr1R Sph1::ura4+ ade6-210 leu1-32 ura4-DS/E his3D	S2
Py 6082	h- JP1611 [pREP81 Leu2+ C-term V5] in sir2Δ::kanMX6 clr4Δ::KanR::clr4+-ade6+ otr1R(SphI)::ura4+ ura4-DS/E ade6-210 leu1-32 his3-D	S2
Py 6085	h- JP1613 [pREP81 Leu2+ Sir2::V5 C-term] in sir2Δ::kanMX6 clr4Δ::KanR::clr4+-ade6+ otr1R(SphI)::ura4+ ura4-DS/E ade6-210 leu1-32 his3-D	S2
Py 7265-7268	h- H3.2K4Q H3.1/H4.1::his3+ H3.3/H4.3::arg3+ JP1326 ade6+ genomic Clr4 (HpaI)::clr4D::KanR his3D arg3D ade6-210 leu1-32 ura4-D18 or DS/E	S5

Py 7190	h- H3.2K4Q H3.1/H4.1::his3+ H3.3/H4.3::arg3+ leu1-32 his3D arg3D ade6-210 ura4-D18?	S5
Py 7216	h- H3.2K4Q H3.1/H4.1::his3+ H3.3/H4.3::arg3+ clr4D::kanR his3D arg3D ade6-210 leu1-32 ura4-D18 or DS/E	S5
Py 7265-7268	h- H3.2K4Q H3.1/H4.1::his3+ H3.3/H4.3::arg3+ JP1326 ade6+ genomic Clr4 (HpaI)::clr4D::KanR his3D arg3D ade6-210 leu1-32 ura4-D18 or DS/E	S5
Py 6394	h- H3.1/H4.1::his3 H3.3/H4.3::arg3 ade6-210 arg3D his3D leu1-32 ura4-DS/E or D18	S6
Py 6358	h- H3.2K14A H3.1/H4.1::his3+ H3.3/H4.3::arg+ clr4D::KanR ade6-210 leu1-32 his3D arg3D ura4D-18 OR DS/E	S6
Py 6486	h- H3.1/H4.1::his3 H3.3/H4.3::arg3 JP1326 ade6+ genomic Clr4 (HpaI)::clr4D::KanR ade6-210 leu1-32? arg3D? his3D?	S6
Py 6495	h- H3.2K14A H3.1/H4.1::his3 H3.3/H4.3::arg3 JP1326 ade6+ genomic Clr4 (HpaI)::clr4D::KanR ade6-210 leu1-32? arg3D? his3D?	S6
Py 6510	h- H3.2K14A H3.1/H4.1::his3 H3.3/H4.3::arg3 JP1326 ade6+ genomic Clr4 (HpaI)::clr4D::KanR ade6-210 leu1-32? arg3D? his3D?	S6
Py 6399	h- ade6-210 leu1-32 ura4-D18 otr1R(SphI)::ura4+ his3D + episomal JP1049 [his3+]	S7
Py 6453	h- clr4D::KanR otr1R(SphI)::ura4+ ade6-210 leu1-32 ura4-DS/E his3D + episomal JP1049 [his3+]	S7,S8
Py 6456	h- clr4D::KanR otr1R(SphI)::ura4+ ade6-210 leu1-32 ura4-DS/E his3D + episomal JP1636 [genomic 3xFlag-clr4 his3+]	S7,S8
Py 8187-8188	h- JP1050 (empty leu+) H3.2K14R H3.1/H4.1::his3+ H3.3/H4.3::arg+ clr4D::KanR ade6-210 leu1-32 his3D arg3D ura4D-18 OR ura4-DS/E	S7
Py 8190-8191	h- JP2100 (genomic Flag-Clr4 leu+) H3.2K14R H3.1/H4.1::his3+ H3.3/H4.3::arg+ clr4D::KanR ade6-210 leu1-32 his3D arg3D ura4D-18 OR ura4-DS/E	S7
Py 8066	h- clr4D::KanR otr1R(SphI)::ura4+ ade6-210 leu1-32 ura4-D18 his3D + episomal JP2111 [genomic 3xFlag-clr4 H410K his3+]	S8
Py 1664	h+/90 clr4::N-term-3XFLAG-clr4 otr1R(SphI)::ura4 ade6-210 arg3D his3D leu1-32 ura4-DS/E	S9
Py 6690-6691	h? clr4::N-term-3XFLAG-clr4 sir2D::kanMX6 otr1R(SphI)::ura4 ade6-210 arg3? his3D leu1-32 ura4-DS/E	S9
Py 42	h- ade6-210 arg3D his3D leu1-32 ura4-D18	S4,S9

Figure S1.

A.

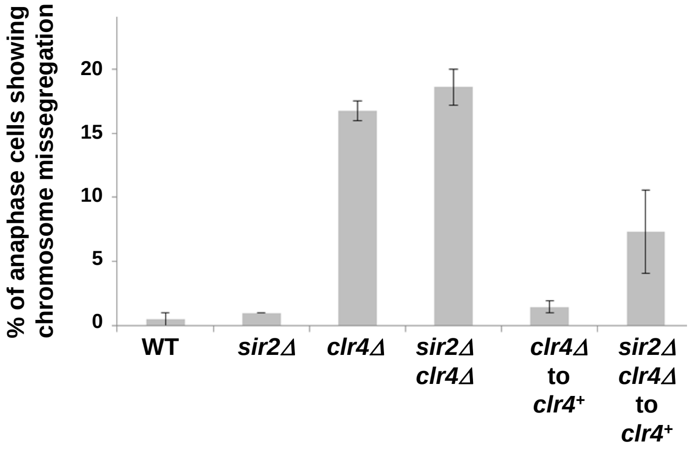
% of anaphase cells showing chromosome missegregation
20
15
10
5
0
WT
sir2Δ
clr4Δ
sir2Δ clr4Δ
clr4Δ to clr4+
sir2Δ clr4Δ to clr4+

Figure S2.

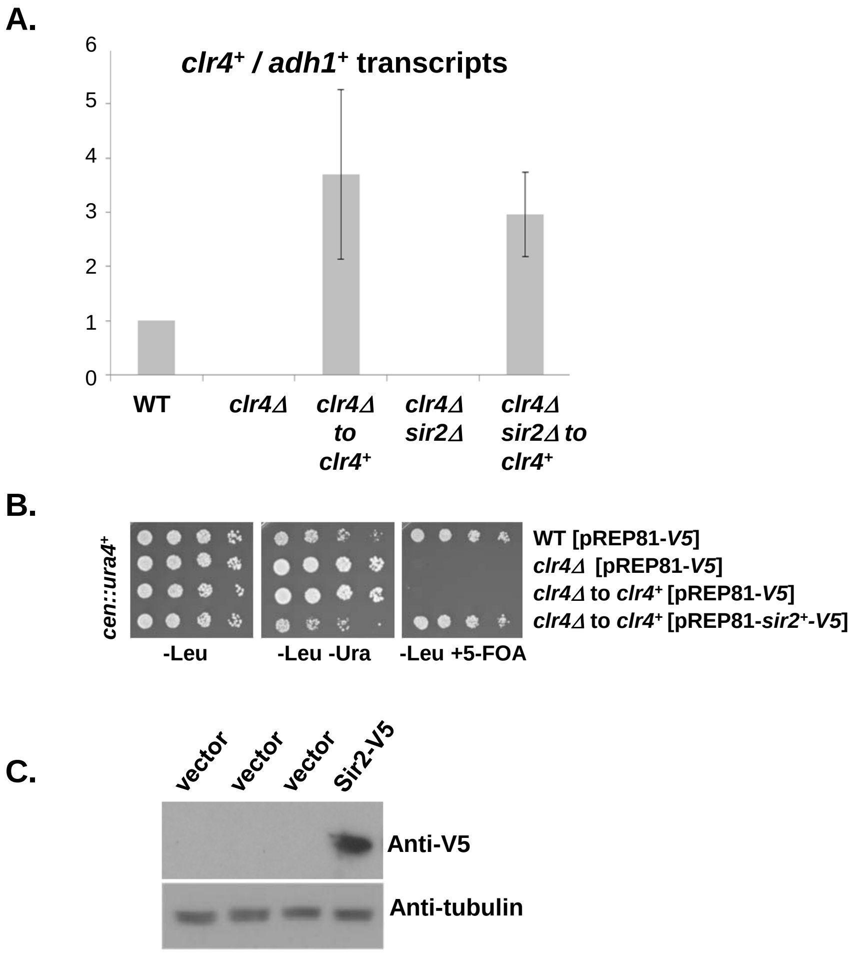
A.
clr4+ / adh1+ transcripts
6
5
4
3
2
1
0
WT
clr4Δ
clr4Δ to clr4+
clr4Δ sir2Δ
clr4Δ sir2Δ to clr4+
B.
cen::ura4+
WT [pREP81-V5]
clr4Δ [pREP81-V5]
clr4Δ to clr4+ [pREP81-V5]
clr4Δ to clr4+ [pREP81-sir2+-V5]
-Leu
-Leu -Ura
-Leu +5-FOA
C.
vector
vector
vector
Sir2-V5
Anti-V5
Anti-tubulin

Figure S3.

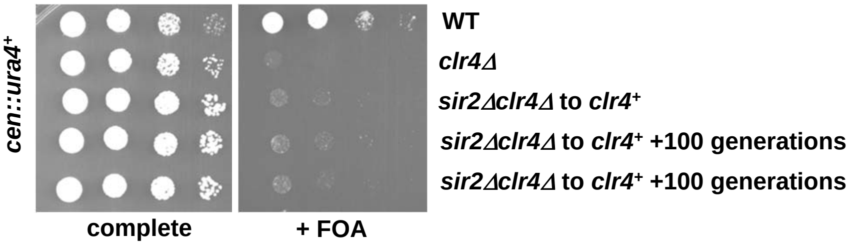
cen::ura4+
WT
clr4Δ
sir2Δclr4Δ to clr4+
sir2Δclr4Δ to clr4+ +100 generations
sir2Δclr4Δ to clr4+ +100 generations
complete
+ FOA

Figure S4.

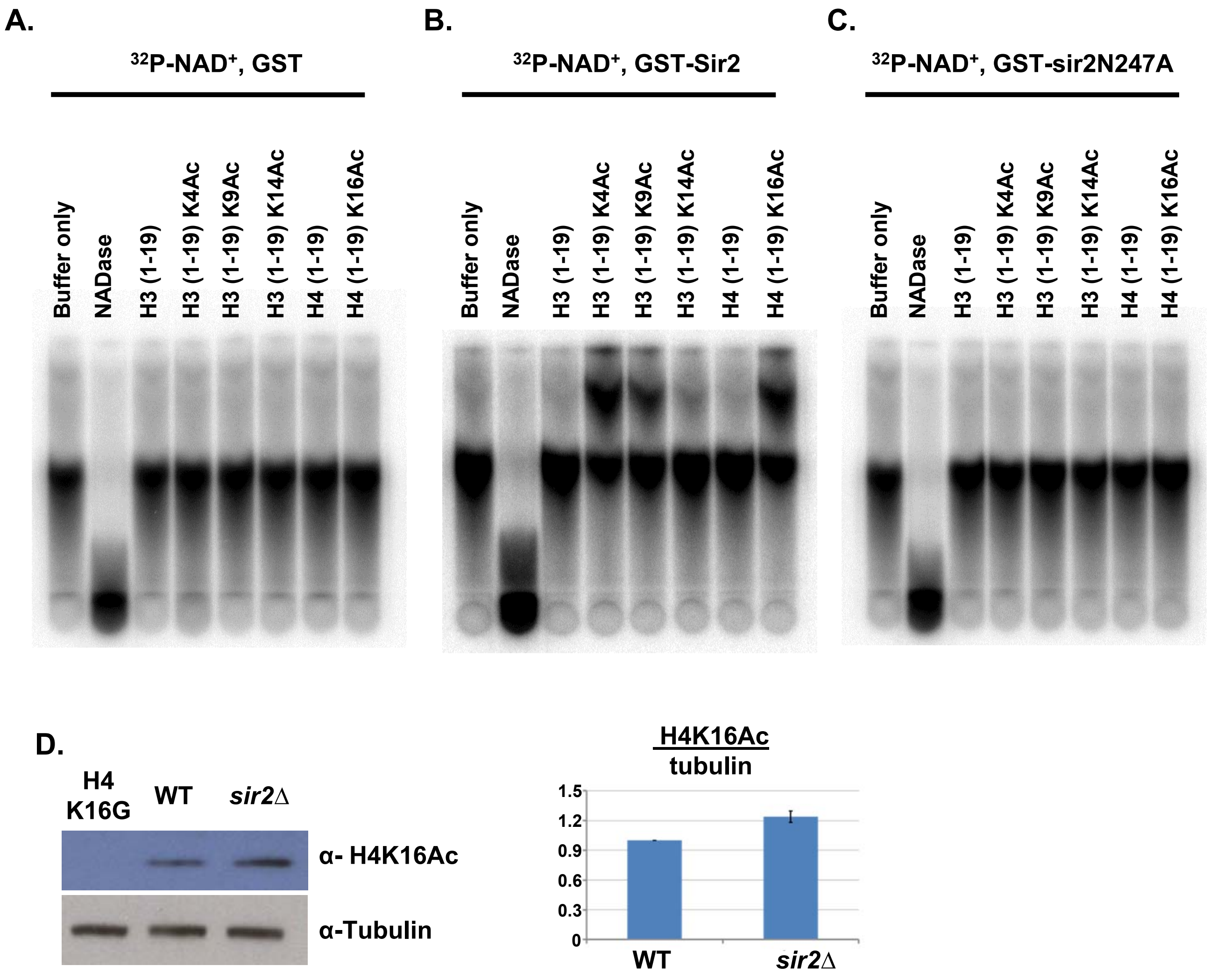
A.
32P-NAD+, GST
B.
32P-NAD+, GST-Sir2
C.
32P-NAD+, GST-sir2N247A
Buffer only
NADase
H3 (1-19)
H3 (1-19) K4Ac
H3 (1-19) K9Ac
H3 (1-19) K14Ac
H4 (1-19)
H4 (1-19) K16Ac
D.
H4 K16G
WT
sir2Δ
α- H4K16Ac
α-Tubulin
H4K16Ac
tubulin
1.5
1.2
0.9
0.6
0.3
0
WT
sir2Δ

Figure S5.

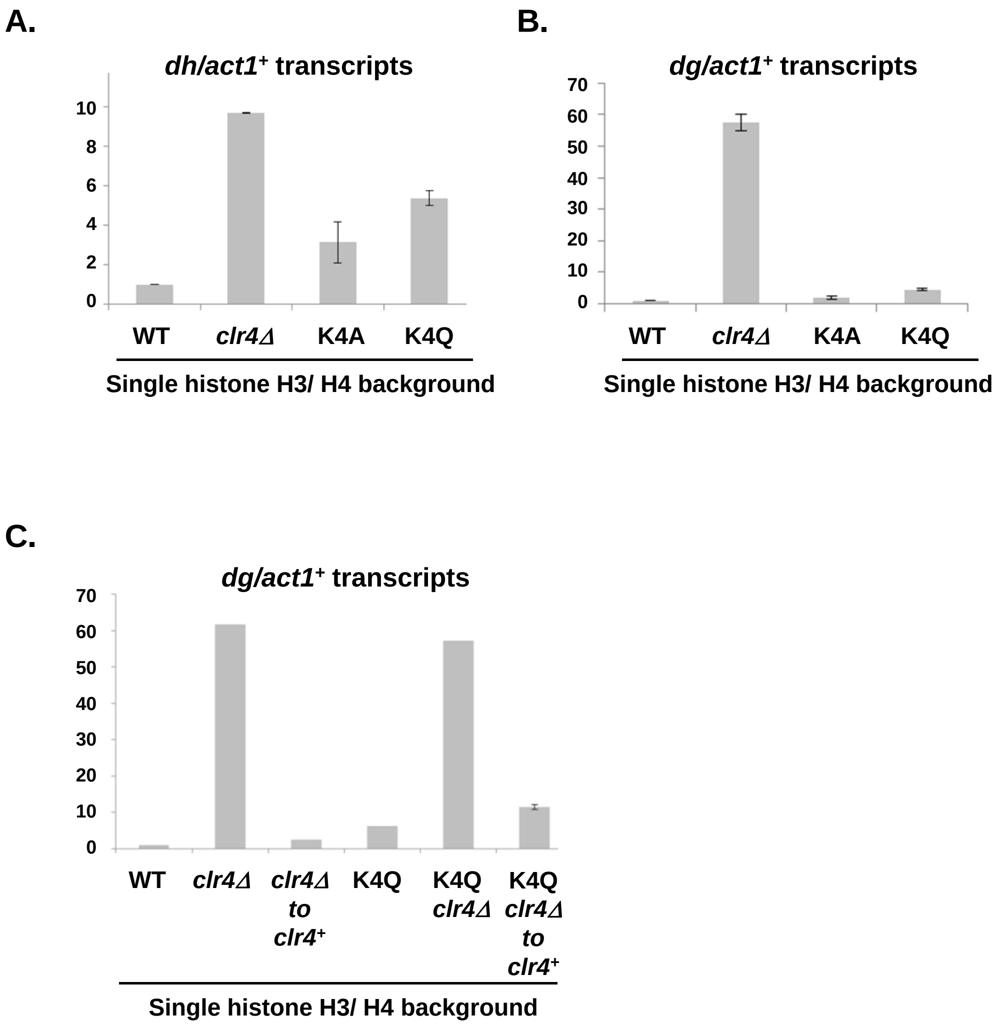
A.
dh/act1+ transcripts
10
8
6
4
2
0
WT
clr4Δ
K4A
K4Q
Single histone H3/ H4 background
B.
dg/act1+ transcripts
70
60
50
40
30
20
10
0
WT
clr4Δ
K4A
K4Q
Single histone H3/ H4 background
C.
dg/act1+ transcripts
70
60
50
40
30
20
10
0
WT
clr4Δ
clr4Δ to clr4+
K4Q
K4Q clr4Δ
K4Q clr4Δ to clr4+
Single histone H3/ H4 background

Figure S6.

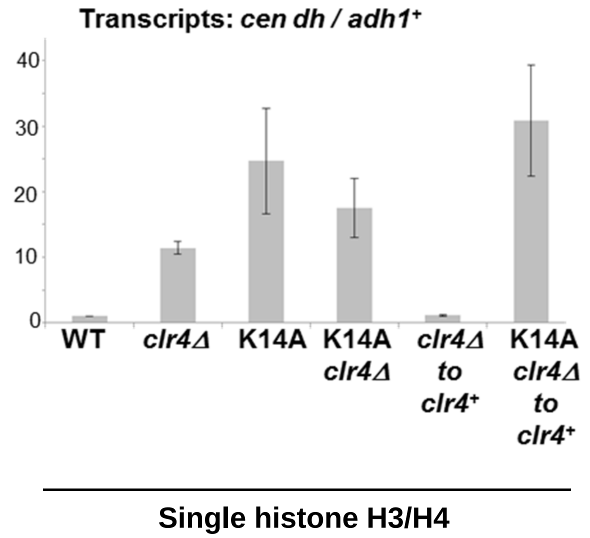
Transcripts: *cen dh / adh1⁺*
40
30
20
10
0
WT
clr4Δ
K14A
K14A *clr4Δ*
clr4Δ to *clr4⁺*
K14A *clr4Δ* to *clr4⁺*
Single histone H3/H4

Figure S7.

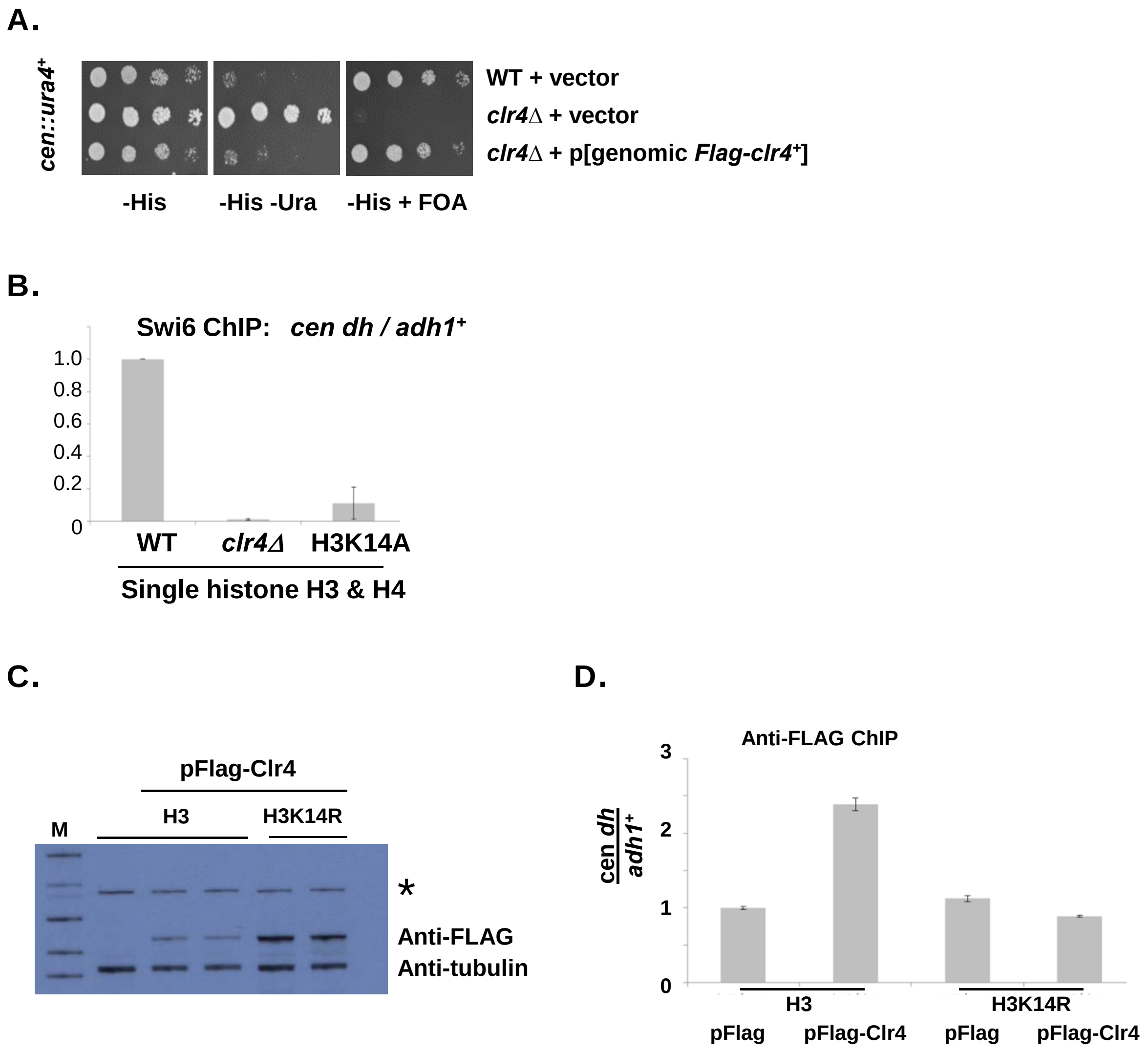
A.
cen::ura4+
WT + vector
clr4Δ + vector
clr4Δ + p[genomic Flag-clr4+]
-His
-His -Ura
-His + FOA
B.
Swi6 ChIP: cen dh / adh1+
1.0
0.8
0.6
0.4
0.2
0
WT
clr4Δ
H3K14A
Single histone H3 & H4
C.
pFlag-Clr4
H3
H3K14R
M
*
Anti-FLAG
Anti-tubulin
D.
Anti-FLAG ChIP
cen dh / adh1+
3
2
1
0
H3
H3K14R
pFlag
pFlag-Clr4
pFlag
pFlag-Clr4

Figure S8.

A.

B.

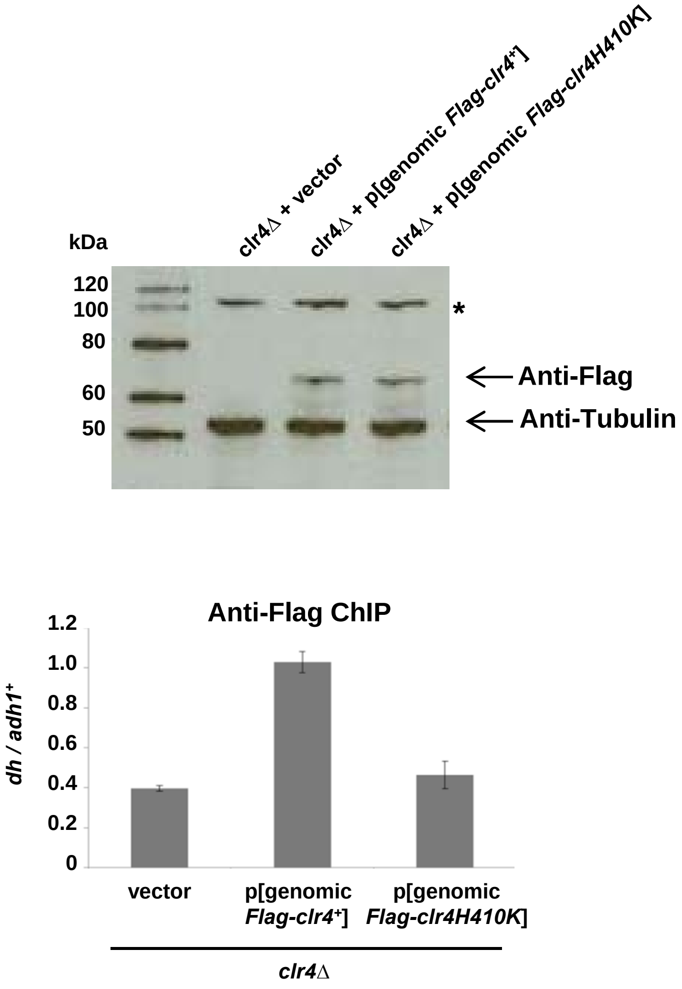

kDa
120
100
80
60
50
clr4Δ + vector
clr4Δ + p[genomic Flag-clr4+]
clr4Δ + p[genomic Flag-clr4H410K]
*
Anti-Flag
Anti-Tubulin
Anti-Flag ChIP
dh / adh1+
1.2
1.0
0.8
0.6
0.4
0.2
0
vector
p[genomic Flag-clr4+]
p[genomic Flag-clr4H410K]
clr4Δ

Figure S9.

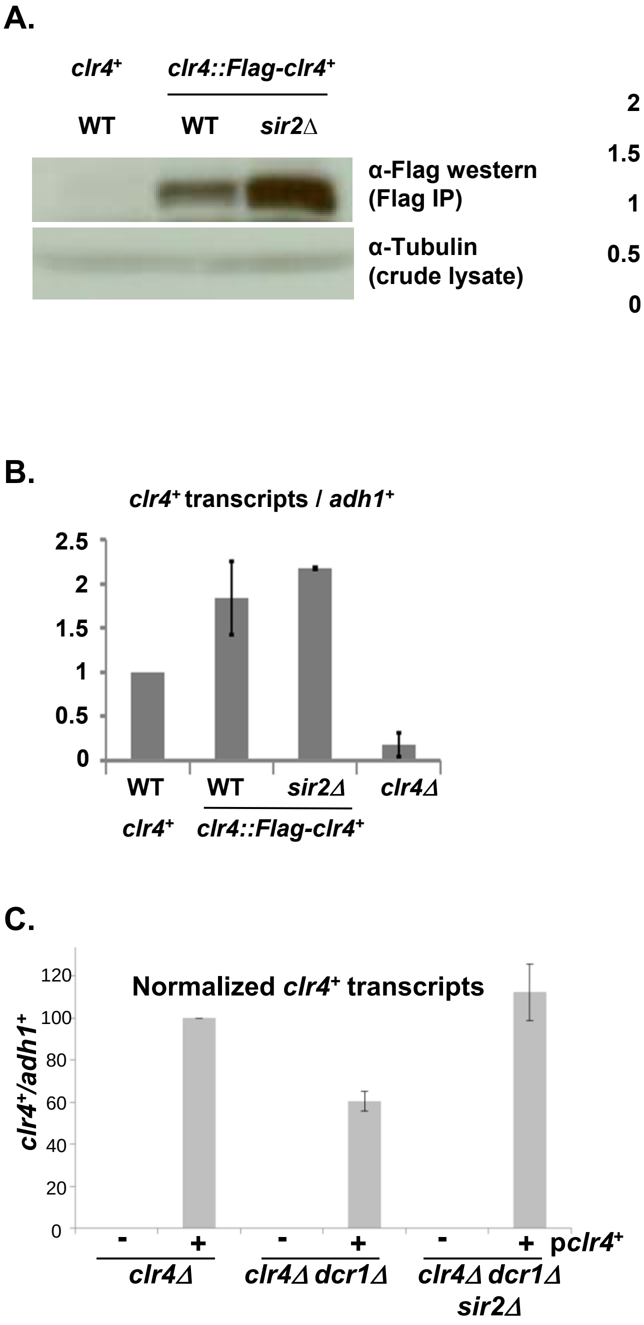
A.
clr4+
clr4::Flag-clr4+
WT
WT
sir2Δ
α-Flag western
(Flag IP)
α-Tubulin
(crude lysate)
Flag-Clr4 / Tubulin
2
1.5
1
0.5
0
WT
sir2Δ
B.
clr4+ transcripts / adh1+
2.5
2
1.5
1
0.5
0
WT
WT
sir2Δ
clr4Δ
clr4+
clr4::Flag-clr4+
C.
Normalized clr4+ transcripts
clr4+/adh1+
120
100
80
60
40
20
0
-
+
-
+
-
+
pclr4+
clr4Δ
clr4Δ dcr1Δ
clr4Δ dcr1Δ
sir2Δ
